# Ischemic Cardiomyopathy versus Non-Ischemic Dilated Cardiomyopathy in Patients with Reduced Ejection Fraction— Clinical Characteristics and Prognosis Depending on Heart Failure Etiology (Data from European Society of Cardiology Heart Failure Registries)

**DOI:** 10.3390/biology11020341

**Published:** 2022-02-21

**Authors:** Agata Tymińska, Krzysztof Ozierański, Paweł Balsam, Cezary Maciejewski, Anna Wancerz, Emil Brociek, Michał Marchel, Maria G. Crespo-Leiro, Aldo P. Maggioni, Jarosław Drożdż, Grzegorz Opolski, Marcin Grabowski, Agnieszka Kapłon-Cieślicka

**Affiliations:** 1 First Department of Cardiology, Medical University of Warsaw, 02-097 Warsaw, Poland; agata.tyminska@wum.edu.pl (A.T.); pawel.balsam@wum.edu.pl (P.B.); cmaciejewski6@gmail.com (C.M.); anna.wancerz22@gmail.com (A.W.); emil.brociek@gmail.com (E.B.); michal.marchel@wum.edu.pl (M.M.); grzegorz.opolski@wum.edu.pl (G.O.); marcin.grabowski@wum.edu.pl (M.G.); agnieszka.kaplon@gmail.com (A.K.-C.); 2 Instituto de Investigaci on Biomedica de A Coruña (INIBIC), Complexo Hospitalario Universitario A Coruña (CHUAC)-CIBERCV, 15006 La Coruña, Spain; marisacrespo@gmail.com; 3 Centro Studi ANMCO (Associazione Nazionale Medici Cardiologi Ospedalieri), 50121 Florence, Italy; maggioni@anmco.it; 4 2nd Department of Cardiology, Central University Hospital, Medical University of Lodz, 92-213 Łódź, Poland; jaroslaw.drozdz@umed.pl

**Keywords:** personalized management, coronary artery disease, atherosclerosis, heart failure, mortality

## Abstract

**Simple Summary:**

Given the high morbidity and mortality linked with heart failure and the need for disease-specific treatment, there is international agreement that there is a significant need for well-planned, large-scale databases showing the true course of heart failure. We present a study based on data from the European Society of Cardiology Heart Failure registries designed to evaluate the prevalence, clinical characteristics, management and outcomes of patients with two main etiologies of heart failure: reduced left ventricular ejection fraction-ischemic cardiomyopathy (ICM) and non-ischemic dilated cardiomyopathy (NIDCM). Our findings show that the patients with ICM were older and had more comorbidities. In contrast, the patients with NIDCM had worse systolic heart function. Apart from the more frequent use of aldosterone antagonists in the NIDCM group, there were no other differences as regards the use of heart failure guideline-recommended medications, implantable cardioverter defibrillators or cardiac resynchronization therapy. One-year prognosis was worse in the ICM patients than in the NIDCM patients. Moreover, ICM etiology itself was associated with a worse one-year outcome.

**Abstract:**

Personalized management involving heart failure (HF) etiology is crucial for better prognoses for HF patients. This study aimed to compare patients with ischemic cardiomyopathy (ICM) and patients with non-ischemic dilated cardiomyopathy (NIDCM) in terms of baseline characteristics and prognosis. We assessed 895 patients with HF with reduced left ventricular ejection fraction participating in the Polish part of the European Society of Cardiology (ESC)-HF registries. ICM was present in 583 patients (65%), NIDCM in 312 patients (35%). The ICM patients were older (*p* < 0.001) and had more comorbidities. The NIDCM patients more frequently had atrial fibrillation (*p* = 0.04) and lower LVEF (*p* = 0.01); therefore, they were treated more often with anticoagulants (*p* = 0.01) and digitalis (*p* < 0.001). The NIDCM patients were prescribed aldosterone antagonists more often (*p* = 0.01). There were no other differences as regards the use of HF guideline-recommended medications, implantable cardioverter defibrillators or cardiac resynchronization therapy. The ICM patients were more likely to be treated with statins (*p* < 0.001) and antiplatelet agents (*p* < 0.001). All-cause death, as well as all-cause death and readmissions for HF at 12 months, occurred more often in the ICM group compared with the NIDCM group (15.9% vs. 10%, *p* = 0.016; and 40.9% vs. 28.6%, *p* = 0.00089, respectively). ICM etiology was an independent predictor of the composite endpoint in the total cohort (*p* = 0.003). The ICM patients were older and had more comorbidities, whereas the NIDCM patients had lower LVEF. One-year prognosis was worse in the ICM patients than in the NIDCM patients. ICM etiology was independently associated with a worse one-year outcome.

## 1. Introduction

Heart failure (HF) incidence and morbidity are on the increase, with a HF prevalence of approximately 1–2% in adults in developed countries, rising to ≥10% in patients aged 70 years or over [1,2]. It is estimated that of those approximately 50% suffer from HF with reduced ejection fraction (HFrEF). Although there are well-established therapies for HFrEF that help to improve symptoms, quality of life and outcomes, the overall prognosis in HF patients remains poor as the 5-year mortality rate after diagnosis is approximately 50% [1,3,4]. It is estimated that around 40% of patients hospitalized for HF will die or will be rehospitalized within a year, with the highest frequency of hospital readmissions in the early post-discharge period [4,5]. Therefore, the current focus of HF therapy is shifting towards a better assessment of the underlying HF etiology and personalized patient management. According to the guidelines of the European Society of Cardiology (ESC), establishing HF etiology should constitute the initial step, crucial for planning an appropriate therapy [1]. However, knowledge about clinical differences and their impact on the prognosis in ischemic cardiomyopathy (ICM) versus non-ischemic dilated cardiomyopathy (NIDCM) patients remains unsatisfactory.

The underlying pathophysiology of both forms of cardiomyopathy is different (i.e., atherosclerosis, inflammation or genetic) and this subsequently may influence the clinical characteristics, the course of the disease and the prognosis, and therefore the management of these patients. Previous studies have reported conflicting results in terms of mortality risk in patients with ICM and NIDCM [6,7,8,9,10]. Patients with ICM are at a greater risk of sudden cardiac death (SCD) than patients with NIDCM; thus, the benefits resulting from implantable cardioverter defibrillators (ICDs) are greater in the first group [1,11]. What is more, a recently published nationwide study has shown one-year mortality to be significantly higher in ICM than in NIDCM patients after ICD implantation for primary prevention of SCD [12]. However, current therapeutic strategies and risk assessments are imperfect, as they are mostly based on the left ventricular ejection fraction (LVEF) and the New York Heart Association Classification (NYHA) and do not account for the underlying HF etiology. Still more data, particularly from real-world patient studies, are necessary to improve etiology-based HF management.

In this study we evaluate the prevalence of ICM and NIDCM etiology as well as the associated clinical characteristics and the prognosis in HFrEF patients.

## 2. Patients and Methods

### 2.1. Study Design

The study involved data from the ESC-HF Pilot and the ESC-HF Long-Term registries of the European Society of Cardiology (ESC). These registries are based on multicenter, prospective, observational surveys lasting from October 2009 to May 2010 in 136 European cardiology centers (including 29 centers in Poland) and from April 2011 to January 2015 in 211 European cardiology centers (including 35 centers in Poland), respectively. Eligible patients were enrolled into the study if they were at least 18 years of age and met the diagnostic criteria for HF, including both outpatients and inpatients with chronic, worsening or new-onset HF. There were no other specific exclusion criteria. The current study included Polish patients of the ESC-HF Pilot Survey and of the ESC-HF Long-Term Registry. The study protocol was approved by the local ethics committees. All participating patients were provided with detailed information and signed informed consent for the study. Records collected in both registries included clinical characteristics, test results, HF management and one-year follow-up. A detailed study design was published previously [13,14].

The current analysis included both ambulatory and hospitalized HFrEF patients (LVEF <40%). The study participants were divided into two groups based on HF etiology —ICM or NIDCM. The ICM group included patients with coronary artery disease as the primary cause of HF, whereas the NIDCM group included patients primarily considered as presenting with dilated cardiomyopathy based on the enrolling investigators’ opinion according to actual knowledge. Patients with a strong etiological factor, i.e., significant valve disease, tachycardia-induced cardiomyopathy and with LVEF ≥ 40% were excluded from the analysis.

### 2.2. Study Endpoints

The primary endpoint was all-cause death at one year. The secondary endpoint was a composite of all-cause death and hospitalization for HF worsening at one year. NYHA class after one year was also analyzed.

Additionally, we sought to determine the clinical characteristics and predictors of one-year outcomes in the studied groups.

### 2.3. Statistical Analysis

The results were presented as medians and quartiles for continuous variables and as frequencies and percentages for categorical and ordinal variables. The frequencies of the categorical and ordinal variables in the groups were compared by Fisher’s exact test and the continuous variables by Mann–Whitney U test, respectively. Kaplan–Meier survival curves were plotted for both study endpoints. Cox proportional hazards regression models were used to identify predictors of the primary and secondary endpoints. Analyses for both study-points regarding HF etiology were performed, adjusting for age, LVEF and NYHA class at baseline, in order to account for important differences in the studied groups. The selection of the potential predictors of outcomes for ICM and NIDCM etiology of HF was guided by background knowledge on the topic. For the selected variables a series of univariable analyses was performed. Variables with the *p*-values below <0.10 threshold in univariable analyses were included in the multivariable analyses. A *p*-value below 0.05 was considered significant for all tests. All tests were two-tailed. Statistical analyses were performed using R software, version 3.6.2 (R Core Team 2020, R Foundation for Statistical Computing, Vienna, Austria).

## 3. Results

### 3.1. Study Group Selection

**Figure 1:**
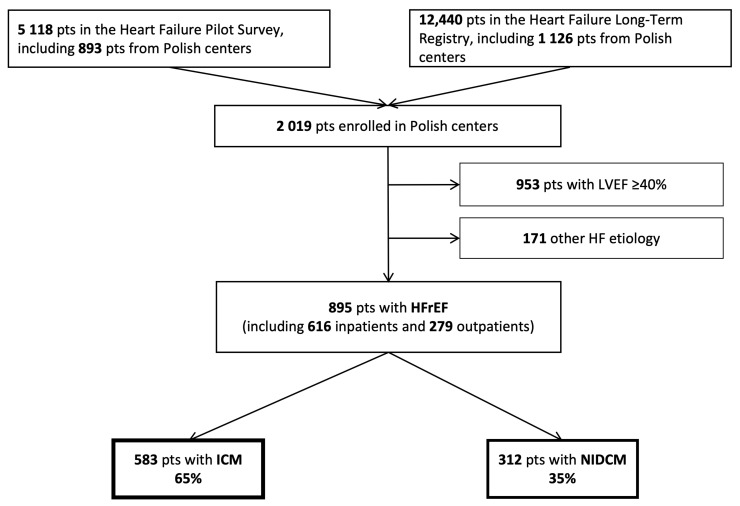
Flow chart of patient enrollment in the current analysis.

Figure 1 shows the flow chart of patient selection for the present study. The ESC-HF Pilot and the ESC-HF Long-Term registries comprised 5118 and 12,440 patients across Europe, respectively. The total Polish cohort of the registry consisted of 2019 patients, including 1415 inpatients and 604 outpatients. Out of them, 895 patients (616 inpatients and 279 outpatients) with HFrEF were included in this study. Among those, 583 (65.1%) and 312 (34.9%) patients had ICM and NIDCM, respectively.

### 3.2. Clinical Characteristics

The ICM patients were older (median: 67 vs. 58 years) and with a higher LVEF (28% vs. 25%) compared with the NIDCM patients. The ICM patients also had more comorbidities (i.e., hypertension, peripheral artery disease, diabetes, chronic kidney disease, chronic obstructive pulmonary disease, previous stroke or transient ischemic attack) than the NIDCM patients. More patients in the ICM group declared current use of tobacco, whereas the NIDCM group more frequently reported alcohol usage. More patients in the NIDCM group had atrial fibrillation; thus, they were more frequently treated with anticoagulants and digitalis. Moreover, the patients with NIDCM received aldosterone antagonists more frequently. There were no other differences as regards the use of HF guideline-recommended medications or ICD/cardiac resynchronization therapy (CRT). Due to the ischemic etiology, the ICM patients were more likely to be treated with statins and antiplatelet agents. Detailed baseline characteristics of the study groups are presented in Table 1 and Table 2.

### 3.3. One-Year Outcomes and Clinical Predictors

**Figure 2:**
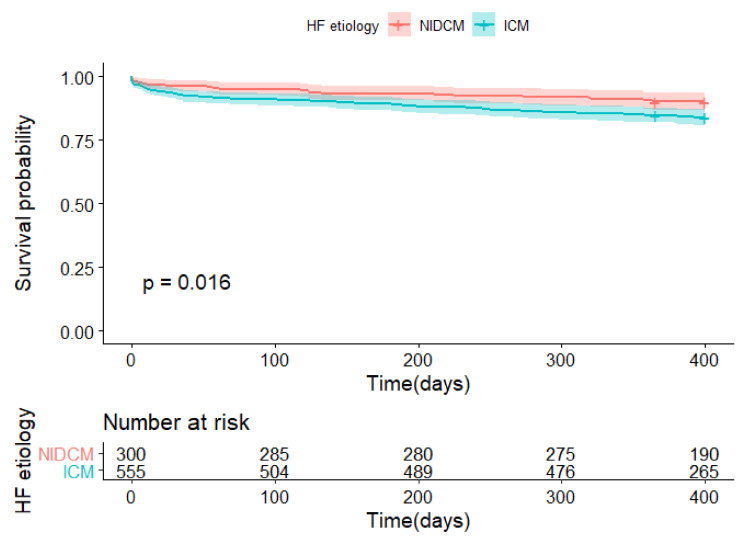
Kaplan–Meier curves for the primary endpoint * of patients with the ICM and NIDCM etiology of HF. ICM—ischemic cardiomyopathy; NIDCM—non-ischemic dilated cardiomyopathy. * Primary endpoint: all-cause death at one year.

**Figure 3:**
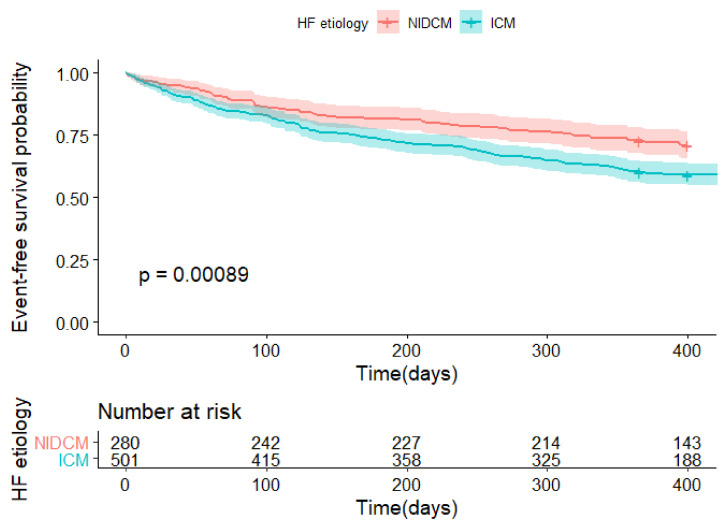
Kaplan–Meier curves for the secondary endpoint* of patients with the ICM and NIDCM etiology of HF. ICM—ischemic cardiomyopathy; NIDCM—non-ischemic dilated cardiomyopathy. * Secondary endpoint: composite of all-cause death and hospitalization for HF worsening at one year).

The primary and secondary endpoints occurred more frequently in the ICM group compared with the NIDCM group (15.9% vs. 10%, *p* = 0.016; and 40.9% vs. 28.6%, *p* = 0.00089, respectively) (Table 1). Kaplan–Meier curves of both study endpoints are plotted in Figure 2 and Figure 3. In addition, Kaplan–Meier curves of both study endpoints after censoring for in-hospital events are included in the Supplementary Materials (Figures S1 and S2). There was no difference between the groups as regards the NYHA class after one-year (*p* = 0.15).

In the multivariable analysis, the ICM etiology was independently associated with the secondary endpoint (1.56 (1.16–2.11), *p* = 0.003) but not with the primary endpoint in the total cohort (Table 3). Independent predictors of the primary and secondary endpoints in both groups are presented in Table 4.

## 4. Discussion

Our study has provided important data on the etiology, associated clinical characteristics and prognoses of real-life patients with HF. The results of this analysis showed that the patients in the ICM group were older and had more comorbidities than the patients in the NIDCM group. The patients with ICM had a higher one-year primary and secondary endpoints occurrence. The ICM etiology was an independent risk factor of the secondary endpoint.

In our study, the ICM etiology was observed in the majority of patients, which is in line with the available data showing ICM as the most common primary etiology of HFrEF, being responsible for 40–70% of cases [1,6]. In clinical practice, it is important to distinguish between ICM and NIDCM because the diagnosis affects management. The management of ICM patients focuses primarily on the evaluation of the extent of CAD and of the possible indications for revascularization. On the other hand, NIDCM may be caused by multiple factors, e.g., toxic or inflammatory (frequently the exact reason remains undetermined) and may require a multidisciplinary approach [1]. In our study, the patients with the ICM etiology were older and had many more underlying chronic diseases (i.e., chronic kidney disease, diabetes, chronic obstructive pulmonary disease) that are pathophysiologically related to the development and complications of diffused atherosclerosis. Nearly the same patient characteristics of both ICM and NIDCM patients were shown in another real-world patient study [12]. Another finding which should be highlighted is that patients from randomized studies had lower ages and rates of comorbidities [15,16,17,18], whereas multimorbidity and older age, being a part of true ICM etiology, significantly influence the clinical presentation and outcomes of patients with HF.

In our study, the ICM patients had significantly worse one-year outcomes than the NIDCM patients. The rates of both all-cause death and all-cause death or HF hospitalization were higher in the ICM than in the NIDCM group, in-line with other real-world data [12], and were higher than in randomized trials [15,16,17,18]. What is more, we observed that the ICM etiology was an independent predictor of the secondary endpoint (all-cause death or HF hospitalization). Other predictors of one-year outcomes in ICM patients were similar to those from the previous analyses. Having examined a group of over 3000 patients with LVEF ≤ 40%, Bart et al. observed that the ischemic etiology of cardiomyopathy is an independent predictor of patient death. The authors also noted that the severity of CAD in coronary angiography had a stronger prognostic value than the etiology of cardiomyopathy itself [19]. Similarly, Stevenson et al. identified CAD itself as an independent predictor of mortality and suggested that patients with ischemic HF should be prioritized for heart transplantation [20]. However, these studies were undertaken over twenty years ago, before the era of life-prolonging treatment (i.e., beta-blockers and renin-angiotensin aldosterone system (RAAS) inhibitors, statins, advanced antiplatelet therapy). The ischemic etiology of HF was also taken into account in the Seattle Heart Failure Model, among various clinical factors used to predict prognosis, mainly in patients with systolic dysfunction [21]. A recently published study showed that ICM etiology adjusted for common risk factors was associated with a higher risk of one- and five-year mortality, as well as HF readmission compared with NIDCM etiology after ICD implantation for primary SCD prevention [12]. It could also be postulated that the extent and severity of CAD offers more prognostic information than the clinical diagnosis of ICM itself. In our previous publication from the ESC-HF Pilot registry on Polish hospitalized patients, we demonstrated that patients with a previous percutaneous coronary intervention (PCI) or coronary artery bypass grafting (CABG) were at a higher risk for death or HF hospitalization than the non-PCI/CABG patients, which could reflect severe CAD and the burden of numerous comorbidities [22].

The NIDCM etiology and its pathophysiology are much more varied and complex than in the case of ICM; therefore, a standard HF pharmacotherapy may not be equally effective (i.e., as in inflammatory cardiomyopathy) [23,24]. However, although the primary mechanism of myocardial injury may be different in patients with ICM and NIDCM, there is an increasing consensus that the progression of HF is directly related to the activation of various neurohormonal systems, particularly the RAAS and the sympathetic nervous systems. It has been demonstrated that proper adherence to guidelines is associated with better survival in patients with HFrEF [25]. Importantly, our patients were treated with a high proportion of life-prolonging HF therapies: 93% vs. 94%, 87% vs. 93% and 74% vs. 82% of patients received beta-blockers, ACE-I/ARB and aldosterone antagonists in ICM and NIDCM, respectively. The patients were treated similarly or even better than in other studies, including randomized trials [18,26,27]. The availability of effective drugs and invasive treatments for ICM should translate into a better prognosis for patients who develop HF due to CAD. Targeted strategies should improve patient care, mainly in ambulatory care, to reduce the rate of readmissions in HF patients.

Electrotherapy with use of ICD and CRT devices is a vital element of HFrEF therapy. ICD reduces the risk of SCD in symptomatic patients with LVEF ≤ 35%, while CRT reduces morbidity and mortality and improves cardiac function and quality of life [1]. However, there are conflicting results on ICD effectiveness in NIDCM patients. Both ICM and NIDCM patients benefit from ICD implantation for primary prevention of SCD due to the reduction of the death rate as compared to conventional drug therapy. Nevertheless, on average, patients with ICM are at a greater risk of SCD than patients with NIDCM [1,28]. Therefore, although the relative benefits are similar, the absolute benefit is greater in patients with ICM [1,11].

Conversely, it is also postulated that the ischemic etiology of HF is less likely to benefit from CRT due to scar tissue [29,30]. However, there is still little evidence that HF etiology affects the effectiveness of CRT and therefore patient outcomes. Current HF ESC guidelines do not account for HF etiology in considerations for CRT implantation [1]. In our study, approximately 30% and 11% of patients in both groups had ICD and CRT, respectively, and the rates were similar or higher than in other observational studies [26,27].

Our study shows that even after considering older age, LVEF and NYHA class on admission, the ICM etiology still carried a worse one-year risk of all-cause death and hospitalization for HF than the NIDCM etiology. Further research is needed to refine the predictors of HF treatment response in the ICM and NIDCM patients so that physicians can more accurately personalize patient management to improve outcomes.

### Limitations

The inclusion of real-life patients followed up by cardiologists is an important advantage of the ESC-HF Pilot and ESC-HF-LT registries. The drawbacks include the partial incompleteness of the data and their observational character. What is more, types of etiology were predefined in the case report form and no specific instructions were provided as to how to identify etiology; therefore, the exact cause of NIDCM or the extent of CAD were unknown. It was also not possible to obtain information on the cause of death (dysrhythmia versus pump failure), which also might have been of interest.

## 5. Conclusions

Results from a real-world HF database of patients followed up by cardiologists showed that ICM was present in the majority of HFrEF patients. The patients with ICM were older and had more comorbidities when compared with the patients with NIDCM. The ICM patients had worse one-year outcomes than the NIDCM patients. The ICM etiology was independently associated with a higher risk of the occurrence of all-cause death or HF hospitalization. The identification of the underlying etiology for HFrEF has significant prognostic and therapeutic ramifications.

## Figures and Tables

**Table 1:** Baseline characteristics and clinical outcomes in total cohort of HF patients and ICM or NIDCM.

	HFrEF Patients (*n* = 895)		*p*-Value
	ICM (*n* = 583)	NIDCM (*n* = 312)	
Baseline characteristics			
Age, years	66.5 (58.7–75.2)	58.2 (49.3–65.2)	**<0.001**
Male	468 (80.3%)	255 (81.7%)	0.66
Previous hospitalization	350 (61.2%); *n* = 572	179 (57.9%); *n* = 309	0.35
BMI, kg/m^2^	27.30 (24.70–30.10); *n* = 554	27.80 (25.00–31.80); *n* = 310	**0.01**
Current LVEF, %	28 (20–33)	25 (20–30)	**0.01**
Previous HF hospitalization	433 (74.3%)	183 (58.7%)	**<0.001**
Prior PCI or CABG	424 (72.7%)	0 (0.0%)	**<0.001**
Moderate or severe mitral regurgitation	302 (57.7%); *n* = 523	149 (54.2%); *n* = 275	0.37
Moderate or severe aortic stenosis	16 (3.1%); *n* = 523	7 (2.6%); *n* = 272	0.83
Moderate or severe aortic regurgitation	41 (7.8%); *n* = 524	13 (4.8%); *n* = 272	0.13
Moderate or severe tricuspid regurgitation	182 (34.8%); *n* = 523	109 (40.1%); *n* = 272	0.16
LVEDD, mm	63.0 (58.0–70.0); *n* = 506	67.0 (60.5–75.0); *n* = 267	**<0.001**
LBBB	89 (17.0%); *n* = 524	59 (21.8%); *n* = 271	0.10
QRS, ms	114.5 (100.0–139.2); *n* = 496	118.0 (100.0–141.0); *n* = 255	0.48
Hypertension	400 (68.7%); *n* = 582	112 (36.1%); *n* = 310	**<0.001**
History of atrial fibrillation	201 (34.5%)	130 (41.8%); *n* = 311	**0.04**
Peripheral artery disease	97 (16.7%); *n* = 582	13 (4.2%)	**<0.001**
Diabetes	233 (40.0%)	82 (26.3%)	**<0.001**
Chronic kidney disease	140 (24.0%)	42 (13.5%)	**<0.001**
COPD	121 (20.8%)	40 (12.8%)	**0.01**
Prior stroke or TIA	78 (13.4%)	24 (7.7%)	**0.01**
Current or former smoking	406 (70.7%); *n* = 574	198 (63.7%); *n* = 311	**0.03**
Alcohol usage	331 (60.5%); *n* = 547	210 (70.7%); *n* = 297	**0.01**
Pacemaker	30 (5.1%)	9 (2.9%)	0.13
ICD	168 (28.8%)	102 (32.7%)	0.25
CRT	61 (10.5%)	36 (11.5%)	0.65
Clinical status and laboratory findings			
Heart rate, b.p.m.	75.0 (67.0–92.0); *n* = 581	80.0 (70.0–97.8)	**0.04**
SBP, mmHg	115 (105–125); *n* = 582	115 (105–130)	0.82
DBP, mmHg	70 (60–80); *n* = 581	70 (70–80)	**<0.001**
NYHA class	*n* = 579	*n* = 312	**0.01**
I	11 (1.9%)	14 (4.5%)	-
II	177 (30.6%)	119 (38.1%)	-
III	254 (43.9%)	117 (37.5%)	-
IV	137 (23.7%)	62 (19.9%)	-
Hemoglobin, g/dL	13.4 (12.2–14.7); *n* = 535	14.2 (13.0–15.2); *n* = 259	**<0.001**
Serum creatinine, mg/dL	1.1 (0.9–1.4); *n* = 550	1.1 (0.9–1.3); *n* = 268	**0.01**
eGFR, mL/min/1.73 m^2^	68.9 (48.1–93.8); *n* = 550	86.3 (60.0–113.7); *n* = 268	**0.01**
Serum sodium, mmol/L	138.8 (136.0–141.0); *n* = 547	139.0 (136.0–141.0); *n* = 263	0.803
NT-proBNP	3566.0 (1575.0–7654.2); *n* = 170	2724.0 (793.0–5227.0); *n* = 97	**0.014**
Pharmacotherapy (at discharge)			
Diuretics	508 (87.3%) *n* = 582	281 (90.4%)	0.19
Aldosterone antagonist	429 (73.7%); *n* = 582	254 (81.7%)	**0.01**
ACE-I	450 (77.3%); *n* = 582	254 (81.4%)	0.17
ARB	54 (9.3%); *n* = 582	35 (11.3%)	0.35
β-blocker	540 (92.8%); *n* = 582	294 (94.2%)	0.48
Statins	492 (84.5%); *n* = 582	154 (49.4%)	**<0.001**
Anticoagulants	213 (36.7%); *n* = 581	146 (46.8%)	**0.01**
Antiplatelets	466 (80.1%); *n* = 582	125 (40.1%)	**<0.001**
Digitalis	135 (23.2%); *n* = 582	112 (35.9%)	**<0.001**
Amiodarone	74 (12.7%); *n* = 582	50 (16.0%)	0.18
Antiarrhytmics	38 (6.5%); *n* = 582	13 (4.2%)	0.17
CCB	50 (8.6%); *n* = 582	9 (2.9%)	**0.001**
One-year outcome			
NYHA	*n* = 447	*n* = 254	0.14
I	37 (8.3%)	35 (13.8%)	-
II	251 (56.2%)	137 (53.9%)	-
III	137 (30.6%)	72 (28.3%)	-
IV	22 (4.9%)	10 (3.9%)	-
Death	88 (15.9%); *n* = 555	30 (10.0%); *n* = 301	**0.02**
Death or rehospitalization	205 (40.9%); *n* = 501	80 (28.6%); *n* = 280	**0.001**

If missing data for the respective variable is present, available cases counts are presented in italics. Bolded text indicates *p* values < 0.05. ACE-I—angiotensin-converting enzyme inhibitor; ARB—angiotensin receptor blocker; BMI—body mass index; b.p.m.—beats per minute; CABG—coronary artery bypass grafting; CCB—calcium channel blocker; COPD—chronic obstructive pulmonary disease; CRT—cardiac resynchronization therapy; DBP—diastolic blood pressure; eGFR—estimated glomerular filtration rate; HF—heart failure; ICD—implantable cardioverter–defibrillator; LBBB—left bundle branch block; LVEDD—left ventricular end-diastolic diameter; LVEF—left ventricular ejection fraction; NIDCM—non-ischemic dilated cardiomyopathy; NT-proBNP—N-terminal pro brain natriuretic peptide; NYHA—New York Heart Association; HFrEF—heart failure with reduced ejection fraction; PCI—percutaneous coronary intervention; SBP—systolic blood pressure; TIA—transient ischemic attack.

**Table 2:** Clinical course of index hospitalization and in-hospital outcomes in HF patients with ICM or NIDCM (only hospitalized participants).

	HFrEF Patients (*n* = 616)		*p*-Value
	ICM (*n* = 433)	NIDCM (*n* = 183)	
Clinical status at hospital admission			
Cardiogenic shock	13/427 (3.1%); *n* = 417	9/175 (5.1%); *n* = 175	0.24
Heart rate, b.p.m.	80.0 (70.0–100.0); *n* = 432	86.0 (73.5–105.0)	**0.01**
SBP, mmHg	120.0 (110.0–140.0); *n* = 432	120.0 (109.5–133.5)	**0.04**
DBP, mmHg	80 (70–84); *n* = 431	76 (70–80)	0.43
NYHA	*n* = 429	*n* = 183	0.96
I	3 (0.7%)	1 (0.5%)	-
II	92 (21.4%)	39 (21.3%)	-
III	201 (46.9%)	83 (45.4%)	-
IV	133 (31.0%)	60 (32.8%)	-
Pacemaker	25 (5.8%)	7 (3.8%)	0.43
CRT	36 (8.3%)	17 (9.3%)	0.75
ICD	112 (25.9%)	52 (28.4%)	0.55
Laboratory findings at admission			
Hemoglobin, g/dL	13.3 (12.1–14.7); *n* = 425	13.9 (12.8–15.1); *n* = 181	**<0.001**
Serum creatinine, mg/dL	1.1 (0.9–1.5); *n* = 428	1.1 (0.9–1.3); *n* = 181	**0.02**
eGFR, mL/min/1.73 m^2^	64.6 (45.6–89.2); *n* = 428	80.5 (58.2–110.7); *n* = 181	**<0.001**
Serum sodium, mmol/L	138.0 (136.0–141.0); *n* = 431	138.0 (136.0–140.5); *n* = 182	0.88
Serum potassium, mmol/L	4.4 (4.1–4.8); *n* = 430	4.5 (4.2–4.8); *n* = 182	0.22
Management during index hospitalization			
Inotropic support	65 (15.0%)	40 (22.0%)	**0.046**
Diuretic i.v.	300 (69.8%); *n* = 430	120 (65.6%)	0.34
Nitrates i.v.	63 (14.6%); *n* = 431	26 (14.2%)	1.0
Clinical status and laboratory findings at discharge			
Heart rate, b.p.m.	70 (65–78); *n* = 420	72 (68–80) *n* = 173	**0.001**
SBP, mmHg	115.0 (105.0–120.0); *n* = 423	115.0 (100.0–120.8); *n* = 176	0.81
DBP, mmHg	70 (60–80); *n* = 422	70 (65–80); *n* = 176	0.13
NYHA	*n* = 424	*n* = 176	**0.04**
I	27 (6.4%)	6 (3.4%)	-
II	219 (51.7%)	111 (63.1%)	-
III	167 (39.4%)	53 (30.1%)	-
IV	11 (2.6%)	6 (3.4%)	-
Hemoglobin, g/dL	12.9 (11.4–14.3); *n* = 279	13.5 (12.6–14.8); *n* = 107	**0.001**
Serum creatinine, mg/dL	1.2 (0.9–1.5); *n* = 329	1.1 (0.9–1.3); *n* = 130	0.07
Serum sodium, mmol/L	138 (136–141); *n* = 351	138 (135–140); *n* = 140	0.34
Serum potassium, mmol/L	4.4 (4.1–4.7); *n* = 352	4.5 (4.2–4.8); *n* = 142	0.13
In-hospital outcomes			
Hospitalization length, days	8 (4–12)	7 (4–12)	**0.49**
Time in ICCU, days	1 (0–5); *n* = 418	0 (0–3.2); *n* = 176	**0.04**
Death during hospitalization	14 (3.2%)	7 (3.8%)	0.81

If missing data for the respective variable is present, available cases counts are presented in italics. Bolded text indicates *p* values < 0.05. ACS—acute coronary syndrome; AF—atrial fibrillation; b.p.m.—beats per minute; CABG—coronary artery bypass grafting; CRT—cardiac resynchronization therapy; DBP—diastolic blood pressure; eGFR—estimated glomerular filtration rate; HF—heart failure; ICCU—intensive cardiac care unit; ICD—implantable cardioverter–defibrillator; ICM—ischemic cardiomyopathy; i.v.—intravenous; NIDCM—non-ischemic dilated cardiomyopathy; NYHA—New York Heart Association; PCI—percutaneous coronary intervention; SBP—systolic blood pressure; VF—ventricular fibrillation; VT—ventricular tachycardia.

**Table 3:** Multivariable analysis of predictors of the primary and secondary endpoints in the total cohort.

	Primary Endpoint			Secondary Endpoint		
Variable	HR	CI	*p*-Value	HR	CI	*p*-Value
HF etiology as ICM (NIDCM as reference)	1.46	0.87–2.47	0.16	1.56	1.16–2.11	**0.003**
Age, years	1.04	1.02–1.06	**<0.001**	1.00	0.99–1.01	0.72
LVEF, %	0.96	0.93–0.98	**0.003**	0.97	0.95–0.99	**<0.001**
NYHA class, * class IV or III vs. II or I	1.66	1.08–2.54	**0.02**	1.72	1.33–2.22	**<0.001**

* At admission to the hospital or first ambulatory visit. Bolded text indicates *p* values < 0.05. ICM—ischemic cardiomyopathy; LVEF—left ventricular ejection fraction; NIDCM—non-ischemic dilated cardiomyopathy; NYHA—New York Heart Association.

**Table 4:** Multivariable analysis of predictors of the primary and secondary endpoints in the ICM and NIDCM groups.

	NIDCM						ICM					
	Primary Endpoint			Secondary Endpoint			Primary Endpoint			Secondary Endpoint		
Variable	HR	CI	*p*-Value	HR	CI	*p*-Value	HR	CI	*p*-Value	HR	CI	*p*-Value
Male sex	-	-	-	-	-	-	0.81	0.48–1.37	0.42	0.72	0.50–1.03	0.07
Age, years	1.03	0.99–1.07	0.17	-	-	-	1.02	1.00–1.05	0.077	-	-	-
BMI, kg/m^2^	-	-	-	-	-	-	0.94	0.89–1.00	0.054	-	-	-
LVEF, %	0.97	0.90–1.04	0.40	0.94	0.90–0.97	**<0.001**	0.96	0.93–0.99	**0.02**	0.98	0.96–1.00	0.08
CABG or PCI in the prior medical history	-	-	-	-	-	-	-	-	-	1.50	1.06–2.14	**0.02**
Peripheral artery disease	-	-	-	-	-	-	1.67	0.97–2.88	0.06	1.35	0.93–1.96	0.11
CKD	2.01	0.72–5.65	0.19	1.92	1.06–3.49	**0.03**	1.48	0.90–2.42	0.12	1.12	0.81–1.55	0.51
Diabetes	-	-	-	-	-	-	-	-	-	1.67	1.24–2.25	**<0.001**
COPD	1.45	0.43–4.94	0.55	-	-	-	1.38	0.83–2.29	0.21	1.20	0.85–1.70	0.30
Heart rate, * b.p.m	1.02	1.005–1.04	**0.011**	1.00	0.99–1.01	0.82	1.00	0.99–1.01	0.41	1.00	1.00–1.01	0.21
SBP, * mmHg	-	-	-	1.00	0.99–1.02	0.72	0.99	0.98–0.99	**0.03**	0.99	0.986–0.999	**0.04**
NYHA class, * class IV or III vs. II or I	6.23	2.02–19.2	**<0.001**	2.02	1.22–3.32	**0.006**	-	-	-	1.50	1.10–2.06	**0.01**
ACE-I	0.71	0.24–2.13	0.54	0.76	0.43–1.36	0.36	0.70	0.42–1.15	0.70	0.69	0.49–0.97	**0.03**
B-blockers	0.12	0.03–0.4	**<0.001**	0.42	0.16–1.12	**0.082**	0.39	0.20–0.74	**0.004**	0.44	0.27–0.73	**<0.001**
MRA	-	-	-	0.91	0.49–1.67	0.76	-	-	-	-	-	-
Diuretics	-	-	-	0.71	0.27–1.90	0.5	-	-	-	-	-	-
Statins	-	-	-	-	-	-	0.67	0.38–1.16	0.15	0.73	0.49–1.08	0.12

* At admission to the hospital or first ambulatory visit. Bolded text indicates *p* values < 0.05. ACE-I—angiotensin-converting enzyme inhibitor; BMI—body mass index; b.p.m.—beats per minute; CABG—coronary artery bypass grafting; CKD—chronic kidney disease; COPD—chronic obstructive pulmonary disease; ICM—ischemic cardiomyopathy; LVEF—left ventricular ejection fraction; NIDCM—non-ischemic dilated cardiomyopathy; MRA—mineralocorticoid receptor antagonist; NYHA—New York Heart Association; PCI—percutaneous coronary intervention; SBP—diastolic blood pressure.

## Data Availability

The data presented in this study are available for three years following the publication on request from the corresponding author.

## Supplementary Materials

The following supporting information can be downloaded at: https://www.mdpi.com/article/10.3390/biology11020341/s1, Figure S1: Kaplan–Meier curves for the primary endpoint* of patients with the ICM and NIDCM etiology of HF after censoring for in-hospital events; Figure S2. Kaplan–Meier curves for the secondary endpoint* of patients with the ICM and NIDCM etiology of HF after censoring for in-hospital events.
