# Supplementary material for: Ischemic Cardiomyopathy versus Non-Ischemic Dilated Cardiomyopathy in Patients with Reduced Ejection Fraction— Clinical Characteristics and Prognosis Depending on Heart Failure Etiology (Data from European Society of Cardiology Heart Failure Registries)
Source: Biology (Basel). 2022 Feb 21;11(2):341. doi: 10.3390/biology11020341 (PMC8869634; doi:10.3390/biology11020341)
Supplement: Supplementary file 1 [file biology-11-00341-s001.zip › biology-1562125-supplementary.pdf]

Figure S1. Kaplan–Meier curves for the primary endpoint* of patients with the ICM and NIDCM etiology of HF after censoring for in-hospital events.

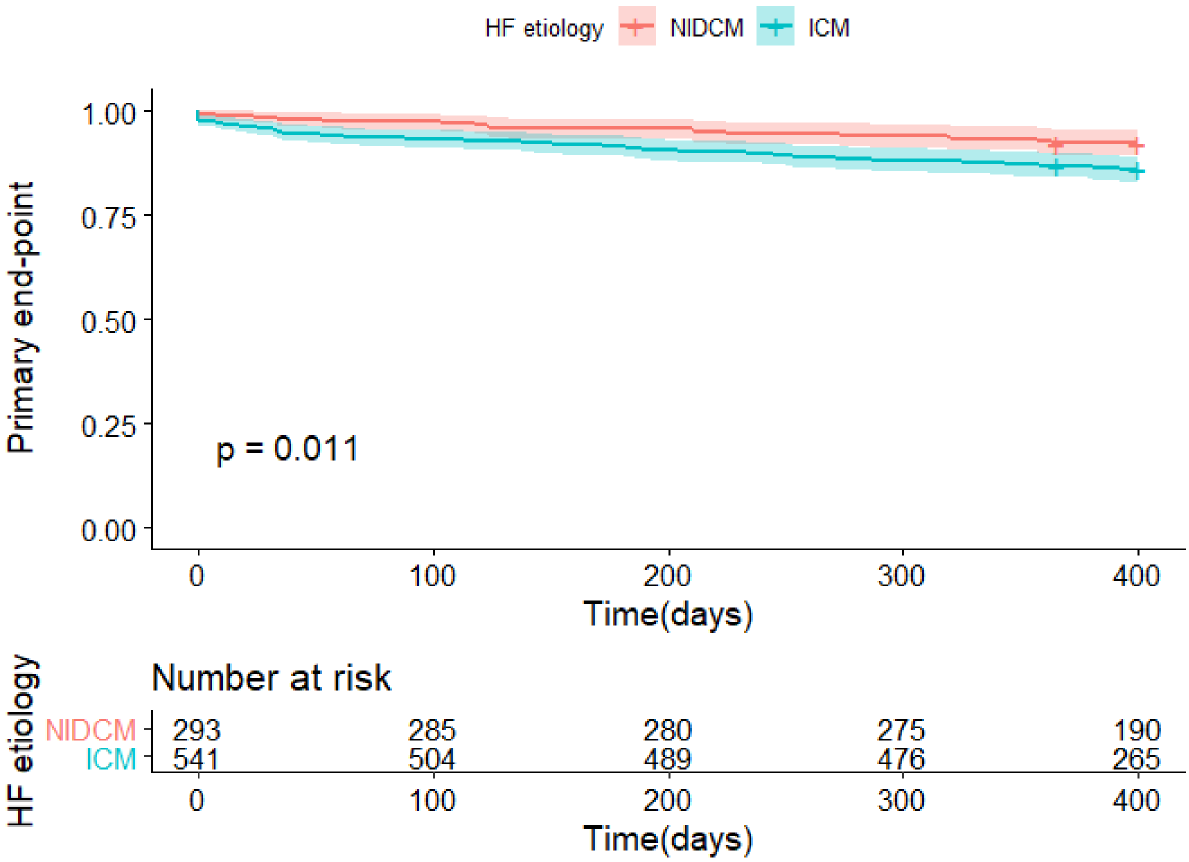

ICM – ischemic cardiomyopathy; NIDCM – non-ischemic dilated cardiomyopathy;
*primary endpoint - all-cause death at one year

Figure S2. Kaplan–Meier curves for the secondary endpoint* of patients with the ICM and NIDCM etiology of HF after censoring for in-hospital events.

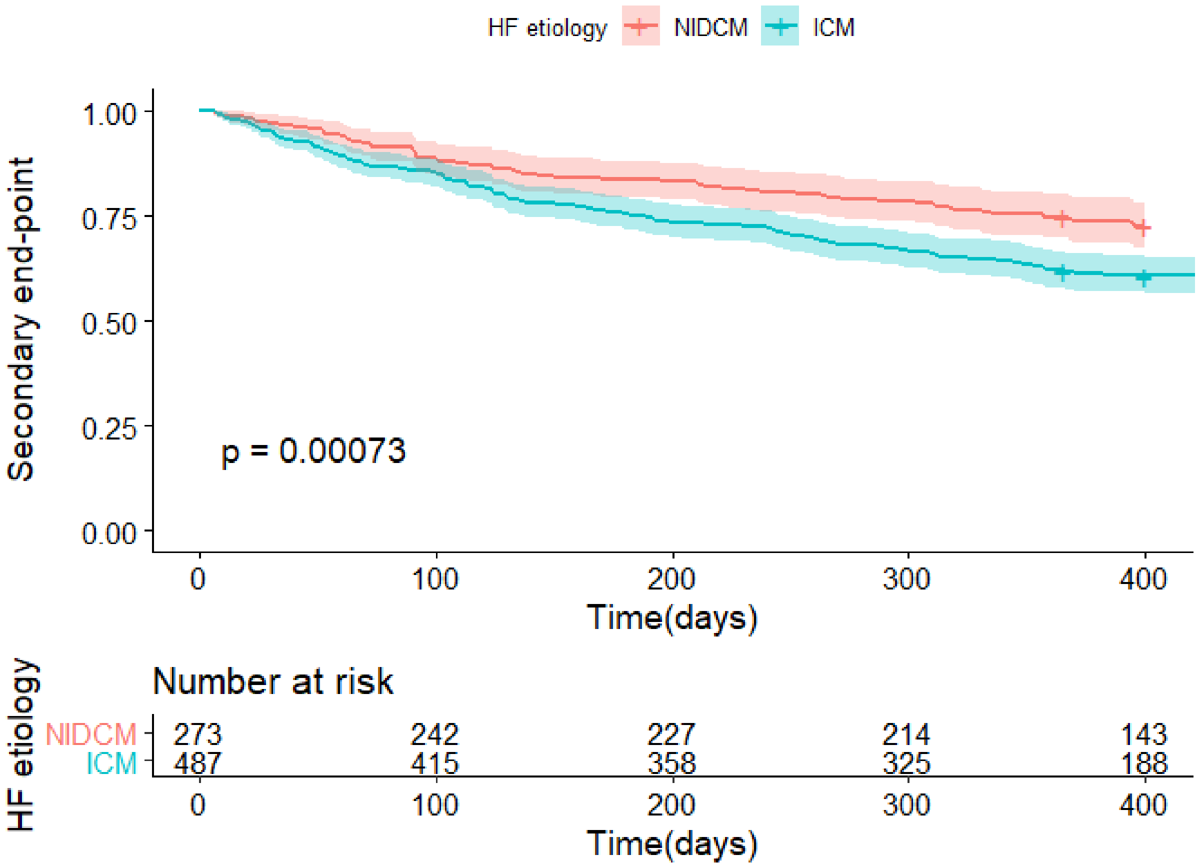

ICM – ischemic cardiomyopathy; NIDCM – non-ischemic dilated cardiomyopathy;
*secondary endpoint - composite of all-cause death and hospitalization for HF worsening at one year)
